# Allometric equations for estimating tree volume and aboveground biomass of *Acacia mangium* Willd. on the Batéké Plateau, Republic of the Congo

**DOI:** 10.1371/journal.pone.0335578

**Published:** 2026-09-03

**Authors:** Chelton Desarmes, Grace Jopaul Loubota Panzou, Flore Hirsch, Paul Bertaux, Nicolas Bayol

**Affiliations:** 1 Société des Plantations Forestières Batéké-Brazzaville (SPF2B), Brazzaville, Republic of the Congo; 2 Forêts Ressources Management (FRM), Montpellier, Hérault, France; 3 Institut Supérieur des Sciences Géographiques, Environnementales et de l’Aménagement (ISSGEA), Université DENIS SASSOU-N’GUESSO, Kintélé, Pool, Republic of the Congo; 4 Faculté des Sciences et Techniques, Laboratoire de Biodiversité, Gestion des Ecosystèmes et de l’Environnement (LBGE), Université Marien NGOUABI, Brazzaville, Republic of the Congo; INRAE, FRANCE

## Abstract

*Acacia mangium* Willd. is a widely planted fast-growing tropical species, but species-specific equations for estimating its tree volume and aboveground biomass remain scarce across tropical Africa. Accurate estimation of these parameters is essential for assessing plantation resources and carbon stocks and for supporting climate-change mitigation initiatives such as Reducing Emissions from Deforestation and Forest Degradation (REDD+). This study developed allometric equations to estimate woody volume and AGB in *A. mangium* plantations on the Batéké Plateau (Republic of the Congo). A total of 54 trees, ranging from 4.5 to 23.8 cm in diameter, were destructively sampled. Trees were measured, felled, and partitioned into stem and large branches (>4 cm), small branches (1–4 cm), phyllodes, and stumps. Subsamples of each compartment were oven-dried and weighed. Results showed that biomass allocation to the stem and large branches increased with tree age, while allocation to small branches and phyllodes declined. A power-law model (Y = a(D²H)ᵇ) provided the best fit (low values of AIC, BIC, RMSE, and bias) for both volume (V = 0.401(D²H)^0.984^) and AGB (AGB = 0.259(D²H)^0.900^). When applied to inventory data from permanent sample plots (trees aged 28–64 months), estimates (mean ± SE) ranged from 38.29 ± 0.90 to 157.62 ± 12.30 m^3^ ha^−1^ for volume and from 29.41 ± 0.70 to 105.51 ± 8.20 Mg ha^−1^ for AGB. These equations provide a robust reference for *A. mangium* on the Batéké Plateau and will strengthen the accuracy of biomass and carbon monitoring for effective forest-based climate mitigation initiatives*.*

## Introduction

Estimating aboveground tree biomass to assess carbon stocks is essential for quantifying the carbon balance in tropical forests. Such estimation has become a global priority, particularly for the implementation of initiatives such as Reducing Emissions from Deforestation and Forest Degradation (REDD+) and Afforestation, Reforestation and Revegetation (ARR) [1,2]. The successful implementation of such initiatives in tropical countries plays a key role in global climate change mitigation, and participating countries must provide detailed strategies and carbon reference levels [3]. However, establishing these reference levels requires accurate estimates of biomass and carbon stocks in developing countries.

Traditionally, allometric relationships have been used to estimate stem volume or tree biomass in forest ecosystems [4]. Allometry describes the relationship between a tree’s measurable characteristics, such as diameter and/or height, and another variable that is more challenging to measure directly, such as biomass or volume [5,6]. Developing allometric models commonly requires destructive sampling, which is costly and logistically demanding [7]. Furthermore, selecting the most appropriate model is essential to improve accuracy [8], yet identifying the best-performing equation is not always straightforward [9].

While volume estimation is critical for forest management and timber commercialization, the demand for biomass estimation has significantly increased over the past decade due to its relevance in climate change mitigation [10]. In Congo Basin countries, the establishment of robust Measurement, Reporting, and Verification (MRV) protocols is hindered by the lack of data necessary for developing allometric equations. This data gap is particularly critical for plantations, which remain among the most effective strategies for mitigating climate change when appropriately designed and managed [11]. Developing region-specific equations can significantly improve biomass estimation and MRV protocols [7].

In the Republic of the Congo, agroforestry and forestry plantations are important components of reforestation, ecological restoration and carbon-sequestration programs [12,13]. They also contribute substantially to wood and energy production, meeting the rapidly growing urban demand for firewood and charcoal [14,15]. In 2013, the government of the Republic of Congo took a proactive approach to afforestation and reforestation, notably through the National Afforestation and Reforestation Program (PRONAR), which aimed to establish 1 million hectares of forest and agroforestry plantations [15–17]. Among the species used, *Acacia mangium* is particularly prominent due to its rapid growth, tolerance of nutrient-poor and acidic soils, and ability to fix atmospheric nitrogen through symbiotic associations with *Rhizobia* bacteria, making it particularly suitable for the long-term success of afforestation initiatives [13,18–21]. It also has high potential for timber and energy production, making it even more attractive for planting programs in tropical areas [13,22,23]. Therefore, the development of allometric equations for volume and aboveground biomass (AGB) for *A. mangium*, represents a key contribution to understanding the carbon cycle. Without locally appropriate equations, plantation wood volume and carbon stocks may be estimated with substantial uncertainty [3]. Although this study focuses on *A. mangium*, the methodological framework (destructive sampling, allometric models, model evaluation) is transferable to other plantation species and regions within the Congo Basin.

In this context, this study aims to develop and test allometric equations for estimating tree volume and aboveground biomass in *A. mangium* plantations on the Batéké Plateau (Republic of the Congo). The specific objectives are to: i) develop allometric equations for estimating woody volume and aboveground biomass at the tree level; and ii) assess the use of these equations with forest inventory data to estimate stand-level volume and AGB in plantations. The resulting equations are intended to support forest inventories, carbon-stock assessment and sustainable management of *Acacia mangium* plantations on the Batéké Plateau and in comparable Central Africa settings.

## Materials and methods

### Study sites

**Fig 1:**
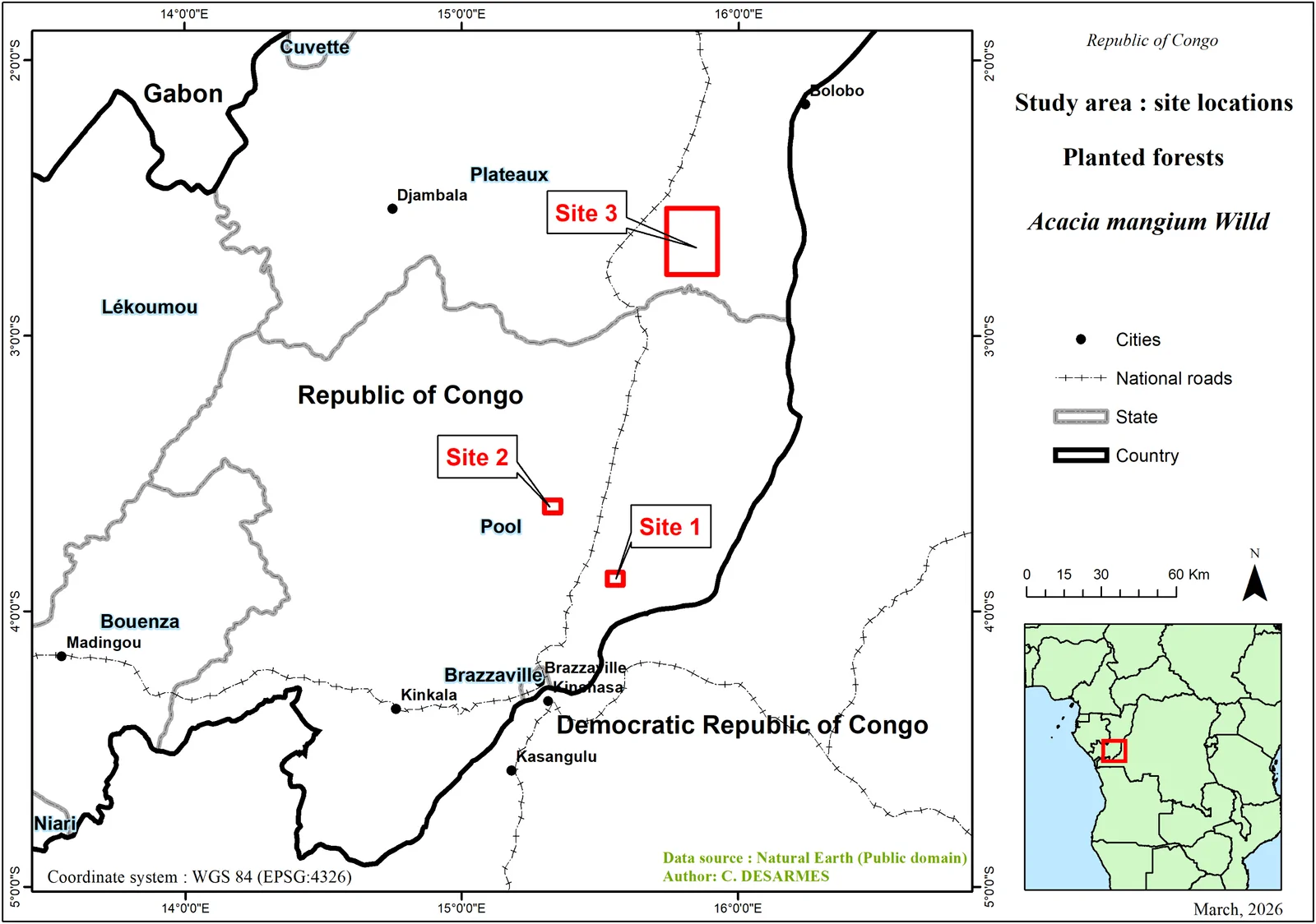
Location of study sites on the Batéké Plateau, Republic of the Congo. The map shows the spatial distribution of the study sites, administrative boundaries, main cities, and road network. Data sources: Administrative boundaries, roads, and populated places from Natural Earth (public domain, https://www.naturalearthdata.com/). Map created by the authors.

This study was conducted in plantations of *Acacia mangium* Willd. (Fabaceae) located on the Batéké Plateau in the Republic of the Congo. The Congolese portion of the Batéké Plateau comprises four distinct savannah sub-plateaus: Koukouya, Djambala, Nsa-Ngo and Mbé/Batéké [24,25]. Fieldwork for this study was carried out in three sites representing three main topographic settings in the Batéké Plateau landscape (Fig 1). The first site (Site 1: 3º52’2”S, 15º31’5”E) was located on the Mbé plateau with a relatively flat terrain at an altitude of 680 m. The second site (Site 2: 3º37’44”S, 15º20’9”E) was located in a transitional area, with an undulating topography, between the Mbé plateau and the hills at an altitude of 700 m. The third site (Site 3: 2º39’27”S, 15º51’6”E) was located in hilly terrain at an altitude of 650 m. All three sites are characterized by tropical a transitional climate with an average annual rainfall of 1500–1800 mm. There is a main dry season from June to September and a shorter one in January and February. The average annual temperature is 25°C.

The soils are developed on sandy parent materials typical of plateau formations such as the Batéké Plateau. According to the FAO World Reference Base for Soil Resources (WRB), these soils are generally classified as Arenosols, locally associated with highly weathered tropical soils such as Ferralsols in some areas [26]. The soils are predominantly ferrallitic, sandy with low clay content, leached with low organic matter and mineral content, classifying them as low fertility soils [24,25]. Previous land use at all sites consisted of open savanna subjected to recurrent annual burning, primarily associated with shifting cultivation practices and hunting activities, which are common land-use systems in Central African savanna landscapes [15]. Prior to planting, soil preparation was limited to mechanical operations, including vegetation clearing and harrowing. No mineral fertilization or liming was applied. Fieldwork was conducted on plantation sites owned and managed by the Société des Plantations Forestières Batéké-Brazzaville (SPF2B). Access to the sites and authorization for destructive sampling were granted by SPF2B. No additional permits were required, as the study was conducted on privately managed land.

### Tree sampling

The sampling was based on pre-existing forest inventory data, comprising 2,722 measurements of diameter at breast height (DBH) collected across the three sites with trees aged 25, 42, and 66 months old. Specifically, Site 1 (66 months old) contributed 85 DBH measurements, Site 2 (25 and 42 months old) contributed 640 measurements, and Site 3 (25 months old) contributed 1,997 measurements. DBH values ranged from 2 cm to 23 cm. Site 3 was subdivided into two sub-units due to pronounced differences in growth observed between two plantation zones, despite their identical age.

The pre-existing DBH measurements data were divided into four classes corresponding to the quartiles of the stand’s DBH distribution (S1 Fig). DBH values below 4 cm were excluded from the sampling frame, as they are younger trees planted to replace dead ones and should have a specific allometric equation [27]. The sampling intensity in term of number of tree for each class was proportionally adjusted on the standard deviation of basal area [6]. A total of 54 trees from all diameter classes and plantation ages were selected in all three sites (Table 1). This number of sampled trees exceeds the recommended minimum (30) for plantation stands [6,28,29] and model precision was subsequently evaluated using confidence intervals and cross-validation.

**Table 1:** Number of trees according to DBH class and plantation age in each site.

DBH classes		Site 1 (plantation age in month)	Site 2 (plantation age in month)		Site 3 (plantation age in month)		Total
		66	42	25	(a) 25	(b) 25	
**1**	**[4; 6.5]**	2	1	1	2	2	8
**2**	**[6.5; 9]**	2	2	1	3	3	11
**3**	**[9; 11.5]**	5	4	3	2	2	16
**4**	**[11.5; DBH**_**max**_ **]**	6	5	2	3	3	19
**Total**		15	12	7	10	10	54

### Tree measurements

Prior to felling, non-destructive tree diameter and height measurements were performed in 2024. The diameter at breast height (DBH) was measured at 1.30 m above the ground using a diameter tape (Richter model: 283D/5) for single-stem trees and multi-stemmed trees (number of stems ranged from 1 to 6). Each stem with a DBH of at least 1 cm at breast height was measured. For each standing tree, total height was measured three times from three different positions using a SUUNTO clinometer (SUUNTO model: PM-5/1520 PC), ensuring that the operator stands at a minimum distance equivalent to the height of the tree to be measured.

Destructive sampling was carried out using a chainsaw. After felling at a stump height of 10 cm, total felled length was measured along the stem with a 20-m measuring tape. Felled trees were cut into four compartments: Stem and large branches (> 4 cm diameter), small branches (1–4 cm diameter), foliage and stump. The stem and large branches were cut into chunks less than 2 m long [6]. The length and diameter at both end of each chunk were measured for each felled tree.

For each compartment, the fresh mass was measured in the field using a 500g precision mechanical balance. Representative samples (trunk: discs of 5 cm at both ends and every 2 m; branches divided into 4 diameter classes: on average, 5 fixed-length samples per class; phyllodes: 3 bags of 10 liters each) were collected and weighed on a 1 g precision balance. These samples were then dried in a circulating air oven at 105°C (for stems, large branches and small branches) or 70°C (for foliage) until their mass stabilized [6,30]. The dry mass of each representative sample was measured using the 1 g precision balance.

For each tree, the observed volume (*V*_*obs*_) was calculated as the sum of the chunks and stump volumes. The volume of each chunk was calculated using Smalian’s formula [31] and the volume of the stump using the truncated cone formula [32]. The observed dry aboveground biomass (*AGB*_*obs*_) was calculated as the sum of the dry aboveground biomass of all the compartments. The dry biomass of each compartment, except the stump, was calculated by multiplying the fresh mass of the compartment by the dry mass/fresh mass ratio determined from the dried samples. The stump biomass was estimated from its volume using the relationship: *AGB*_*stump*_ *= V*_*stump*_ * d, where d is the anhydrous density (0.507 g/cm^3^) of *Acacia mangium* [33].

### Data analysis

To develop the allometric equations, both non-destructive measurements (diameter at breast height, *D*, and total height, *H*) and the destructive measurements (observed volume, *V*_*obs*_, and aboveground biomass, *AGB*_*obs*_) were used (S1 Dataset). Allometric models using D as a predictor have been widely used in allometric studies due to their simplicity and operational applicability [7,13,30,34]. However, integrating total height with diameter (*D²H*) in the allometric models can significantly improve the accuracy of woody volume and AGB estimates [8,35–37]. Since tree height measurements are difficult in tropical forests [38,39], we first evaluated the absence of significant differences between non-destructive tree height measurements and destructive measurements on felled trees. Given the strong correlation (Pearson’s r = 0.98, p-value < 0.001) and no significant difference between the non-destructive and destructive height measurements (p-value > 0.05; repeated measures analysis of variance; S1 Table and S2 Fig), the non-destructive height measurement can be confidently used to establish volume and AGB allometric equations.

For multi-stemmed trees, an equivalent diameter was calculated from individual stem diameters as the square root of the sum of squared diameters (D = √(∑Di²)) where D is the stem diameter. This corresponds to the diameter of a single stem with the same total basal area as all stems combined [40].

For each predictor (D in cm or D^2^H in m^3^), four allometric models (linear, power, exponential and logarithmic) commonly used worldwide to estimate volume or aboveground biomass were tested [34,41]. The modelling approach used the following equation based on the diameter (D) and total height (H) of a tree *i* belonging to all three sites:


$$\begin{array}{c} Y_{i}=a\times D_{i}+b+\varepsilon_{i}\;or\;Y_{i}=a\times {D_{i}}^{\text{2}}\times H_{i}+b\;+\varepsilon_{i}\text{ linear model} \end{array}$$ (1)

$$\begin{array}{c} Y_{i}=a\times {D_{i}}^{b}\;+\varepsilon_{i}\;or\;Y_{i}=a\times {\left({D_{i}}^{\text{2}}\times H_{i}\right)}^{b}\;+\varepsilon_{i}\text{ power model} \end{array}$$ (2)

$$\begin{array}{c} Y_{i}=a\times e^{b\times D_{i}}\;+\varepsilon_{i}\;or\;Y_{i}=a\times e^{b\times ({D_{i}}^{\text{2}}\times H_{i})}+\varepsilon_{i}\text{ exponential model} \end{array}$$ (3)

$$\begin{array}{c} Y_{i}=a\times \text{ln}\left(D_{i}\right)-b\;+\varepsilon_{i}\;or\;Y_{i}=a\times \text{ln}\left({D_{i}}^{\text{2}}\times H_{i}\right)-b\;+\varepsilon_{i}\text{ logarithmic model} \end{array}$$ (4)

where a and b are the fitted coefficient or exponent, $Y_{i}$ is alternatively the observed tree volume (V_obs_ in m^3^) or the aboveground biomass (AGB_obs_ in Mg) and $\varepsilon_{i}$ is the error.

For the power and exponential models, initial parameter values were derived from linear regressions on log-transformed data. These starting values were then used to fit the final models directly to the untransformed data using the “nls()” function, thereby avoiding correction of retransformation bias [42,43].

From woody volume, tree AGB can be also estimated using the biomass expansion factor (BEF) which represents the part of biomass contained in the small branches (D < 4 cm) and phyllodes [44]. The biomass expansion factor (BEF) was defined as the ratio of total reference AGB to the dry biomass corresponding to the reference woody volume: BEF = AGB_obs_/(V_obs_), where V_obs_ comprised the stump, stem, and branches (≥ 4 cm in diameter) and ρ (0.507 Mg m^−3^) was wood density of *A. mangium* [33]. BEF was estimated as the slope of a linear regression of AGB_obs_ on V_obs_ fitted through the origin.

Model selection was based on the following criteria [6,45,46]: (a) the coefficient of determination (R²), representing the proportion of explained variance; (b) model performance indicators, including root mean square error (RMSE) and prediction bias; (c) Akaike’s Information Criterion (AIC), and Bayesian Information Criterion (BIC) which balances model accuracy and complexity. A lower AIC or BIC value indicates a better model fit.

To evaluate the predictive performance of the selected models, a leave-one-out cross validation (LOO-CV) was used to calculate the mean absolute error (MAE), the mean absolute relative error (MARE), and the mean prediction bias of the models. Each tree was omitted in turn, the model was refitted to the remaining 53 trees, and the omitted response was predicted [47–49]. Site effects were also assessed using likelihood-ratio tests between nested models, combined with parametric bootstrap (999 simulations) to account for the non-linear mixed-effects structure. Bootstrap samples were generated under the null hypothesis, and p-values were estimated as the proportion of simulated likelihood-ratio statistics exceeding the observed value [50,51].

To describe variation in model-derived stand estimates among the three age classes, forest inventory data were converted into volume and AGB using the best fitted models. The forest inventory data consisted of eight permanent sample plots (PSP), with two PSP established in Site 1 (64 months old) and six in Site 2 (40 months old for 4 PSP and 28 months old for 2 PSP). Each PSP corresponds to three lines of thirty spots, i.e., approximately 0.0907 ha. The tree density was estimated at approximately 715 trees/ha in the Site 1 and 880 trees/ha in the Site 2. The forest inventory data (DBH and H) for all living trees (a total of 634 trees) in the PSP was collected in March 2024. These forest inventory data were used to assess the impact of these equations for plantation ages ranging from 28 to 64 months. A linear regression was fitted between the estimated volume or AGB, and plantation age to assess the validity of allometric equations across the plantation age range (25–66 months).

All statistical analyses were performed using the R environment, version 4.4.2 (S1 Code) [52]. Graphical outputs were generated using the *ggplot2* package [53]. Residual normality and homoscedasticity were assessed graphically and using the Shapiro–Wilk and Breusch–Pagan tests, respectively. Model outputs and statistical analyses were conducted using the *broom* and *rstatix* packages [54–56]. Statistical significance was determined at P < 0.05.

## Results

### Overview of sampled trees

The 54 sampled trees had a mean diameter at breast height (DBH) of 11.0 ± 4.5 cm and a mean height (H) of 10.3 ± 3.8 m (Table 2). DBH and H ranged from 4.5 to 23.8 cm and 5.3 to 20.2 m, respectively, indicating the range of validity of the allometric models. The observed woody volume and aboveground biomass (AGB) of felled trees ranged from 0.007 to 0.477 m^3^ (mean ± SE = 0.073 ± 0.091 m^3^) and from 0.004 to 0.284 Mg (mean ± SE = 0.052 ± 0.059 Mg), respectively (Table 2).

**Table 2:** Mean ± standard deviation of the tree measurements (DBH, total height, volume, and AGB) in each study site.

Site	Plantation age (month)	Mean DBH (cm)	Mean height (m)	Mean volume of felled tree (m^3^)	Mean AGB of felled tree (Mg)
**Site 1**	66	12.7 ± 5.7	14.6 ± 3.8	0.135 ± 0.142	0.089 ± 0.093
**Site 2**	42	12.0 ± 4.4	10.3 ± 1.6	0.041 ± 0.027	0.032 ± 0.021
	25	9.9 ± 2.8	8.4 ± 1.4	0.070 ± 0.055	0.050 ± 0.036
**Site 3**	(a) 25	9.7 ± 4.4	9.2 ± 1.8	0.048 ± 0.051	0.038 ± 0.039
	(b) 25	9.5 ± 3.3	6.3 ± 1.1	0.030 ± 0.027	0.028 ± 0.023
**mean**	40.2	11.0 ± 4.5	10.3 ± 3.8	0.073 ± 0.091	0.052 ± 0.059

**Fig 2:**
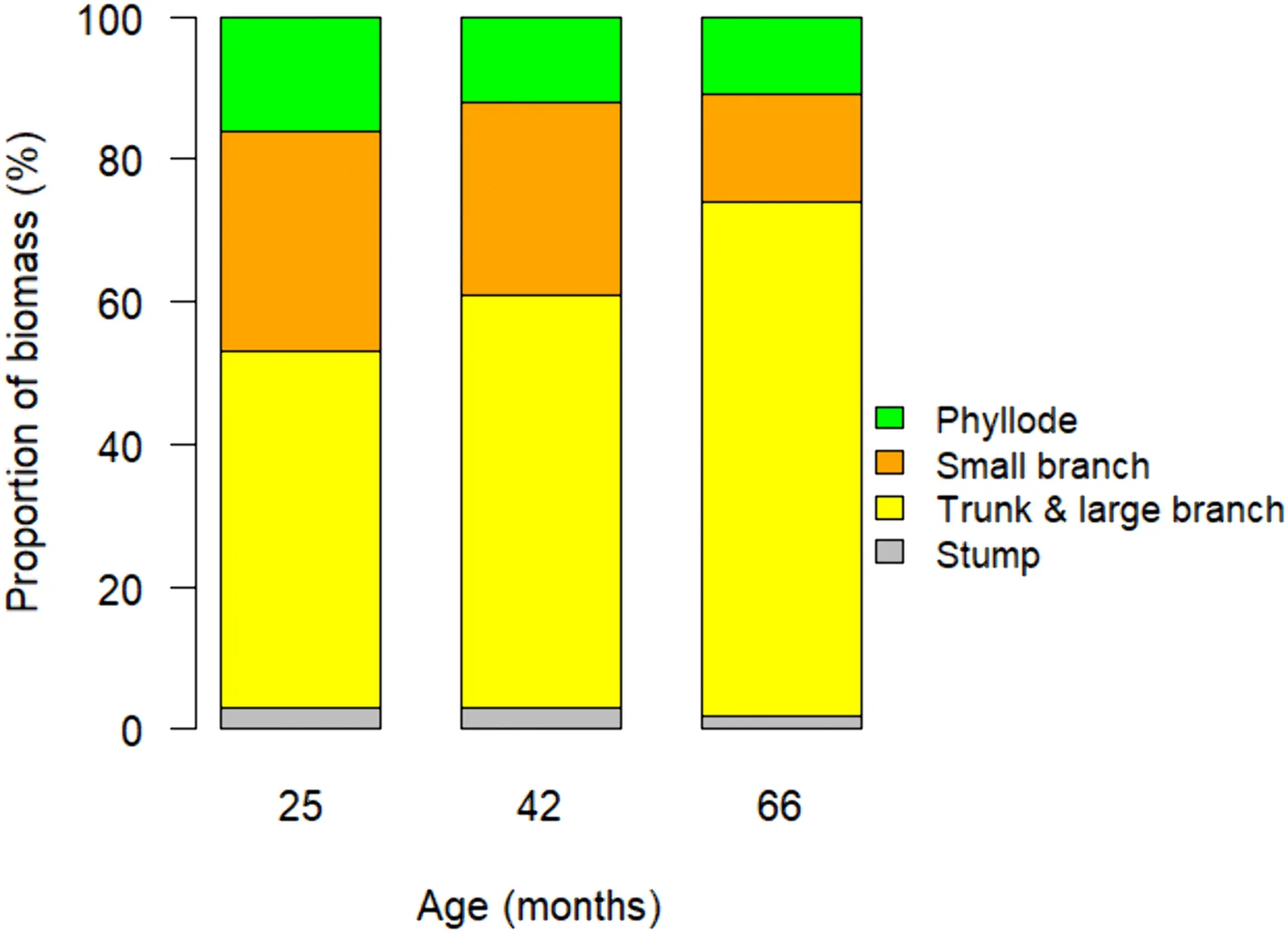
Distribution of the AGB allocation according to the compartments (Trunk and large branch, small branch, phyllode and Stump) and plantation ages.

The allocation of AGB among tree compartments varied with plantation age (Fig 2). Between 25 and 66 months, the proportion of AGB allocated to the trunk and large branches increased, whereas the proportions allocated to small branches and phyllodes decreased. Consistent with this shift in biomass allocation, the biomass expansion factor (BEF) decreased with plantation age (Table 3), from 1.57 ± 0.04 at 25 months to 1.36 ± 0.06 at 42 months and 1.28 ± 0.05 at 66 months. The coefficients of determination for the age-specific BEF regressions ranged from 0.977 to 0.983. When all 54 trees were analyzed together, the pooled regression yielded a BEF of 1.32 ± 0.03 (R² = 0.974).

**Table 3:** Biomass Expansion Factor (BEF) by stand age and for the global model.

Stand age (months)	n	BEF (± SE)	R²
25	27	1.57 ± 0.04	0.983
42	12	1.36 ± 0.06	0.978
66	15	1.28 ± 0.05	0.977
All ages combined	54	1.32 ± 0.03	0.974

Estimate BEF ± standard error (SE), number of observations (n), coefficient of determination (R²) reported for stand age.

### Allometric models for volume and aboveground biomass

Models using diameter and height as predictors exhibited better performance than those using only diameter, except for the exponential model (Table 4). Among the tested models, the power model with D²H as predictor (Model 4: *Y = a(D²H)ᵇ*) demonstrated the best overall performance for both volume and AGB allometric equations. For woody volume, however, Model 2 (linear) and Model 4 (power) showed comparable performance. Their respective goodness-of-fit statistics were: R² = 0.98 for both models; AIC = −310.89 and −310.80; BIC = −304.92 and −304.83; RMSE = 0.012867 m^3^ and 0.012878 m^3^. Model 4 nevertheless showed a lower relative bias than Model 2 (−1.29% versus −3.98%). Leave-one-out cross-validation also produced similar mean absolute errors for the two models (0.0080 and 0.0081 m^3^, respectively), while the mean absolute relative error was lower for Model 4 than for Model 2 (12.28% versus 13.45%). Mean prediction biases were −0.0001 m^3^ for Model 2 and −0.0005 m^3^ for Model 4.

**Table 4:** Allometric equations for woody volume (V) and aboveground biomass (AGB).

Models		Coefficient and exponent		Performance criteria				
**N°**	**Equation**	**a**	**b**	**R²**	**AIC**	**BIC**	**RMSE**	**Bias (%)**
1	*V = aD + b*	0.01785	−0.12377	0.79	−184.40	−178.43	0.041507	40.83
	*AGB = aD + b*	0.01191	−0.07889	0.83	−243.14	−237.17	0.024094	33.23
2	*V = a(D²H) + b*	0.40053	0.00167	0.98	−310.89	−304.92	0.012867	−3.98
	*AGB = a(D²H) + b*	0.25869	0.00630	0.97	−333.17	−327.20	0.010469	−12.48
3	*V = aD* ^*b*^	0.00005	2.89288	0.92	−234.45	−228.49	0.026112	5.28
	*AGB = aD* ^*b*^	0.00007	2.60588	0.93	−291.51	−285.54	0.015396	3.09
4	*V = a(D²H)* ^*b*^	0.40115	0.98435	0.98	−310.80	−304.83	0.012878	−1.29
	*AGB = a(D²H)* ^*b*^	0.25917	0.90017	0.97	−335.49	−329.53	0.010245	−3.66
5	*V = ae* ^*b D*^	0.00753	0.17491	0.92	−234.26	−228.30	0.026158	−33.43
	*AGB = ae* ^*b D*^	0.00676	0.16063	0.92	−283.48	−277.51	0.016584	−30.54
6	*V = ae* ^*b(D²H)*^	0.04470	2.18624	0.85	−202.16	−196.19	0.035214	−102.42
	*AGB = ae* ^*b(D²H)*^	0.03350	2.04148	0.82	−238.80	−232.84	0.025081	−87.28
7	*V = aln(D) – b*	0.18079	−0.34663	0.63	−154.84	−148.87	0.054576	42.87
	*AGB = aln(D) – b*	0.12180	−0.23032	0.68	−208.92	−202.95	0.033076	39.46
8	*V = aln(D²H) – b*	0.07286	0.24022	0.72	−168.65	−162.69	0.048022	56.12
	*AGB = aln(D²H) – b*	0.04797	0.16247	0.74	−219.03	−213.06	0.030120	37.69

For each model, the estimated coefficients (a, b) and performance criteria are reported, including coefficient of determination (R²), Akaike Information Criterion (AIC), Bayesian Information Criterion (BIC), root mean square error (RMSE), and bias (%). Volume is expressed in cubic meters (m^3^), AGB in megagrams (Mg), D in centimeters (cm), and D²H in cubic meters (m^3^).

For aboveground biomass, Model 4 clearly outperformed the competing equations, with the highest coefficient of determination (R² = 0.97), the lowest AIC (−335.49) and BIC (−329.53), and the lowest RMSE (0.010245 Mg) and relative bias (−3.66%). Its predictive performance was further characterized by a leave-one-out cross-validation MAE of 0.0076 Mg, a MARE of 19.08%, and a mean prediction bias of −0.0001 Mg. Based on these combined goodness-of-fit and cross-validation statistics, the power model using D²H was retained for subsequent woody volume and AGB estimations.

**Fig 3:**
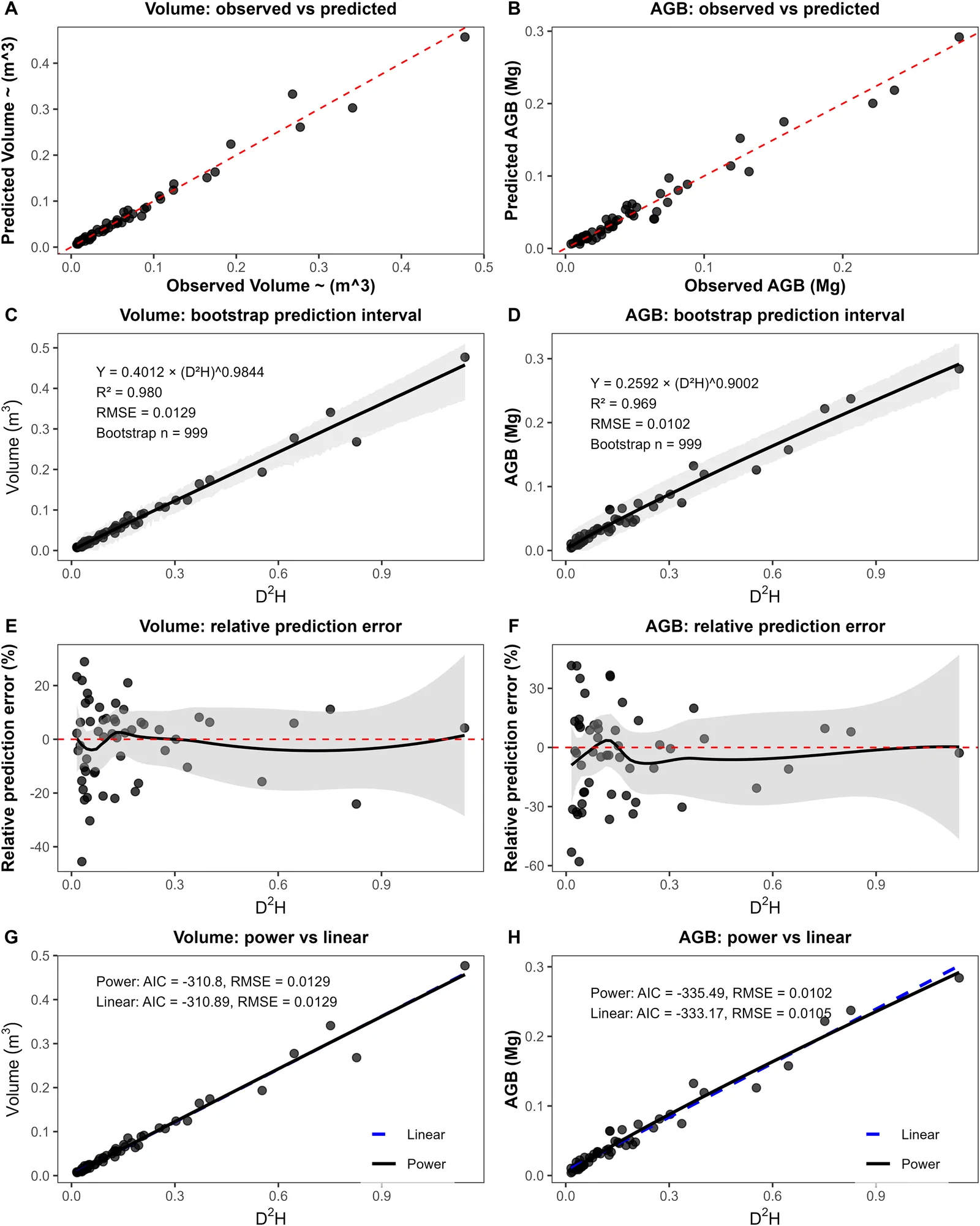
Validation and performance assessment of the selected allometric models. (A and B) observed versus predicted values, (C and D) prediction intervals, (E and F) relative prediction error across the predictor range, and (G and H) comparison between power-law and linear models.

For each best model parameter, the confidence intervals (95%) were estimated. For tree volume, the scaling coefficient *a* was 0.401 (95% CI: 0.336–0.444), and the allometric exponent *b* was 0.984 (95% CI: 0.868–1.040). For AGB, *a* was estimated at 0.259 (95% CI: 0.230–0.284), while *b* was 0.900 (95% CI: 0.837–0.972). Fig 3 summarizes the predictive performance of the selected models by showing observed versus predicted values relative to the 1:1 line, prediction intervals, relative prediction errors across the D²H range, and comparisons between the power and linear model forms (Fig 3). The relative prediction errors shown in Fig 3E–F were generally distributed around zero over most of the sampled D²H range, indicating no strong systematic tendency toward over or underprediction.

### Site effects on the selected allometric models

For tree volume, introducing a random site effect on the scaling parameter *a* did not improve model fit (LR < 0.001, bootstrap p = 0.834). Allowing the allometric exponent *b* to vary among sites also produced no significant improvement (LR = 0.274, bootstrap p = 0.198). These results indicated no detectable site-related variation in the volume-D²H relationship. For AGB, inclusion of a random site effect on the scaling parameter *a* significantly improved model fit (LR = 0.755, bootstrap p = 0.030), whereas allowing the exponent *b* to vary among sites did not provide an additional significant improvement (LR = 2.782, bootstrap p = 0.063). Site-specific estimates of *a* for AGB ranged from 0.244 at Site 2 to 0.282 at Site 3b (Table 5), while the common exponent *b* was estimated at 0.897 (95% CI: 0.852–0.992). Relative to the global scaling estimate of 0.259, site-specific values varied from approximately −6% to +9%, indicating that the magnitude of site-related variation was limited.

**Table 5:** Site-specific estimates of parameters and confidence intervals for the selected AGB power model.

Site	Scaling parameter *a*	*95%* Confidence interval
Site 1	0.260	0.238-0.285
Site 2	0.244	0.222-0.313
Site 3a	0.275	0.239-0.338
Site 3b	0.282	0.251-0.392

### Application of volume and AGB allometric models

**Fig 4:**
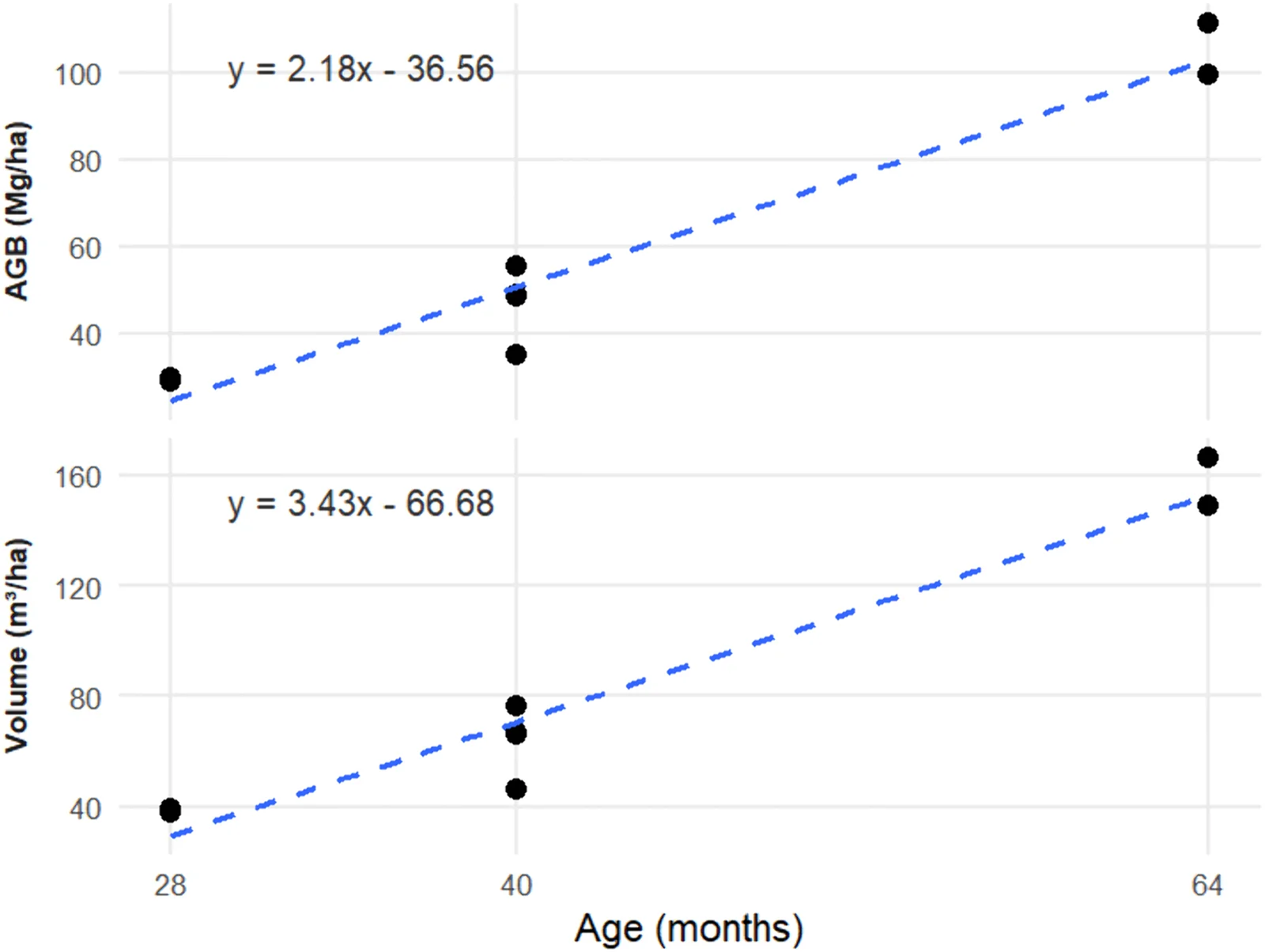
Stand-level woody volume and AGB of 8 PSP located on Site 1 and 2.

The power model (model 4) was the best model to estimate stand-level volume and AGB. Using data from the eight permanent sample plots from 28 to 64 months, our results showed a progressive increase in stand-level volume and AGB with plantation age (Fig 4). The volume (mean ± SE) increased from 38.29 ± 0.90 m^3^ ha^−1^ at 28 months to 157.62 ± 12.30 m^3^ ha^−1^ at 64 months, with a mean annual increment ranging from 16.41 m^3^ ha^−1^ year^−1^ to 29.55 m^3^ ha^−1^ year^−1^. Similarly, AGB increased from 29.41 ± 0.70 Mg ha^−1^ at 28 months to 105.51 ± 8.20 Mg ha^−1^ at 64 months, with a mean annual increment ranging from 12.60 Mg ha^−1^ year^−1^ to 19.78 Mg ha^−1^ year^−1^.

## Discussion

This study developed species-specific equations for estimating tree volume and aboveground biomass (AGB) in young *Acacia mangium* plantations established on the Batéké Plateau. Power functions based on D²H provided reliable estimates of both response variables, although the linear and power models performed almost identically for tree volume. Volume allometry was consistent among sites, whereas the scaling coefficient of the AGB equation varied significantly but moderately among sites. The magnitude of variation in parameter *a* for AGB remained relatively limited (approximately ±7% around the global estimate), suggesting that the practical gain from using site-specific equations may be moderate. While site-specific models may improve accuracy for local applications, the global model remains a robust and operationally efficient alternative, particularly for large-scale or multi-site implementations. Moreover, age and site were partly confounded because the oldest trees were sampled at Site 1, whereas Sites 2 and 3 mainly represented younger stands. The observed site effect on AGB may consequently reflect a combination of edaphic conditions, stand age, growth history and tree architecture.

Biomass allocation also changed with plantation age: the proportion allocated to trunks and large branches increased, while the biomass expansion factor (BEF) decreased. Application of the selected equations to permanent sample plots showed increasing stand volume and AGB between 28 and 64 months.

These findings should be interpreted within the limits of the sampling design. The destructive sample comprised 54 trees covering DBH values from 4.5 to 23.8 cm and plantation ages from 25 to 66 months. The equations should therefore not be extrapolated beyond these diameter and age ranges without additional validation. Finally, thinning, pruning, fertilization, spacing and other silvicultural treatments were not experimentally evaluated, so the equations are most directly applicable to plantations managed under conditions comparable to those examined here.

### Aboveground biomass partitioning and BEF

Destructive sampling revealed that the trunk and large branches accounted for the majority of aboveground biomass (> 50%) across all plantation ages, consistent with findings for *A. mangium* in Indonesia [44,57] and *A. mearnsii* in Ethiopia [58]. The increasing contribution of these woody compartments from 25 to 66 months was accompanied by declining proportions of small branches and phyllodes, reflecting an age-related shift from crown development toward stem wood accumulation. Comparable ontogenetic changes have been reported in Indonesian *A. mangium* plantations, where stem biomass increased and leaf biomass declined with stand development and competition [44,57].

The pooled BEF estimate of 1.32 was very close to the mean value of 1.332 reported by Miyakuni et al. [44] for 3- to 8-year-old *A. mangium* stands in West Java. However, the age-specific results indicate that BEF should not be considered constant: it decreased from 1.57 at 25 months to 1.28 at 66 months in the present study, while Miyakuni et al. reported a further decline to 1.18 in a 10-year-old stand. The use of age-class-specific BEF values is therefore regarded as a relevant approach to improve the accuracy of biomass estimates and to reduce uncertainties in carbon stock assessments. The BEF estimates obtained in both studies were substantially lower than the default BEF value of 3.4 (range: 2.0–9.0) recommended by the Intergovernmental Panel on Climate Change (IPCC) guidelines for tropical forests [59]. Because this IPCC value is a broad forest-type default rather than a species-specific coefficient, its application to young, even-aged *A. mangium* plantations could substantially overestimate AGB. BEF is known to vary with multiple factors, including forest type, tree age, stand density, growth conditions, and climate [35].

As highlighted by Miyakuni [44] and further supported by the present study, no statistically robust relationships could be established between BEF and commonly used predictors such as D, H, or D²H (S3 Fig). This lack of predictive consistency underscores the empirical nature of BEF and suggests that its estimation should rely on direct measurements rather than on allometric surrogates.

### Silvicultural influences on biomass dynamics

Silvicultural practices can alter both the magnitude and allocation of plantation biomass and may consequently affect the transferability of allometric equations among management regimes. In *Acacia mangium* plantations, initial spacing influences individual-tree growth and the proportion of dry biomass allocated to the stem, whereas thinning and stand development have been associated with differences in wood density and in the relative allocation of biomass to stems and foliage [57,60]. Pruning modifies crown structure and stem form by removing branches and may therefore alter the contribution of crown biomass to total AGB and potentially the biomass expansion factor, although its direct effect on BEF has not yet been quantified for this species [61]. Fertilization can also modify biomass production and allocation: phosphorus application in *A. mangium* increased diameter, stand volume, aboveground and root biomass, while reducing the root-to-shoot ratio and disproportionately increasing stem biomass [62]. Rotation age further determines standing biomass at harvest and interacts with the intended production objective, as biomass allocation and the energetic value of the harvested material change during stand development [57,60].

Evidence from Indonesian *A. mangium* plantations showed differences in wood density and biomass partitioning between thinned and unthinned stands, as well as age-related increases in the proportion of stem biomass and decreases in foliage biomass [57]. Mixed plantations of *A. mangium* and eucalypts in Brazil and the Republic of Congo also demonstrated that biomass production depends on the balance between interspecific competition, resource complementarity and nitrogen-related facilitation. In the Congolese experiment, these interactions resulted in stemwood mean annual increments that were 17–34% higher in some mixtures than in the eucalypt monoculture at harvest, whereas equivalent gains were not consistently observed at the Brazilian sites [63,64]. These findings suggest that equations developed in untreated monocultures should be validated before being transferred to thinned, fertilized, pruned or mixed-species plantations, particularly when high-precision biomass or carbon estimates are required.

### Allometric equations for volume and AGB estimation

The selection of an appropriate allometric model is a critical step in the estimation of forest aboveground biomass [65,66]. As shown in this study and in previous work, the inclusion of additional predictors in combination with tree diameter provides better estimates of tree volume and aboveground biomass [6,67]. The power model using D²H as predictor showed the best overall performance for both volume and AGB allometric equations. These findings indicate that D²H provides a more accurate representation of the variability in observed tree volume and aboveground biomass. Previous studies have also shown that power functions were able to estimate AGB with a high goodness of fit [68]. Power functions represent most commonly used functional form in allometric equations for estimating the tree volume and aboveground biomass in Central Africa [67,69,70], and used to describe the allometric relationships for a wide range of plant species [71]. Power function models also express the allometry between different parts of the plant, such as the proportionality in the relative increments between stem biomass and girth of the trees [6].

The relative prediction errors were generally centered around zero across the sampled D²H range for both volume and AGB (Fig 3E-F). The volume model had a MARE of 12.28% and a mean prediction bias of −0.0005 m^3^, whereas the AGB model had a MARE of 19.08% and a mean prediction bias of −0.0001 Mg. These near-zero mean biases indicate no marked systematic over- or underprediction, although individual-tree predictions were less precise for AGB than for volume. Because LOOCV evaluates predictions for observations excluded from model fitting, these results provide a direct assessment of model predictive performance [47].

### Applications across carbon, industrial and land-use contexts

Reliable volume and AGB equations have applications extending beyond the estimation of standing carbon stocks. In industrial plantations, woody volume equations provide estimates of total and merchantable wood volume, thereby supporting forest inventories, yield assessment, harvest planning, and the estimation of feedstock available for pulpwood and other wood products [21,72]. *Acacia mangium* is widely cultivated for short-rotation fibre, pulp, solid wood, fuelwood, charcoal, construction materials, agroforestry, and the rehabilitation of degraded land [21,73]. In bioenergy systems, total AGB and compartment-specific estimates can be used to quantify potentially recoverable stem wood, bark, treetops, and other harvesting residues. Experimental work in Brazil showed that *A. mangium* harvest residues, including wood, bark, and treetops, had suitable physical and energetic properties and could be used either directly or after densification into briquettes [74]. A complementary study found that retaining small proportions of bark with *A. mangium* wood was technically feasible and improved several energetic properties of the resulting biomass [75].

### Stand-level volume and AGB accumulation on the Batéké Plateau

Using the allometric models developed in this study, estimated values ranged from 38.29 ± 0.90 m^3^ ha^−1^ to 157.62 ± 12.30 m^3^ ha^−1^ for volume and from 29.41 ± 0.70 Mg ha^−1^ to 105.51 ± 8.20 Mg ha^−1^ for AGB in *A. mangium* plantations of 28, 40 and 64 months on the Batéké Plateau. These results show that the both volume and AGB estimates of *A. mangium* increased with stand age, consistent with previous results in Indonesia [57], in Colombia [76] and in Vietnam [77]. These findings, as well as other studies [78,79], suggest that stand age is a key driver of ecosystem carbon dynamics and provide important insights into carbon sequestration processes. However, direct comparison of stand-level values must account for differences in planting density, survival, site fertility, provenance, management history and the definition of included biomass compartments.

## Conclusion

This study provides a new destructive dataset for *Acacia mangium* Willd. plantations on the Batéké Plateau, covering the early growth stages (25–66 months). Based on 54 destructively sampled trees, power equations using D²H as predictor provided the most reliable estimates of woody volume and AGB. Application to stands aged 28–64 months yielded estimates up to 157.62 m^3^ ha^−1^ of volume and 105.51 Mg ha^−1^ of AGB at 64 months. These locally calibrated equations provide robust tools for forest inventories, carbon accounting and MRV, yield and harvest planning, bioenergy resource assessment, and the evaluation of plantation contributions to wood production, climate-change mitigation and degraded-land restoration in Central Africa.

## Supporting information

S1 Fig The stand’s DBH distribution of pre-inventory data. (TIF)

S2 Fig Comparison of standing-tree and felled-tree height measurements. (TIF)

S3 Fig Relationship between BEF and D, H and D²H. (TIF)

S1 Dataset Data underlying the results presented in this article. (XLSX)

S1 Table Comparison of tree-height measurement methods using repeated-measures ANOVA. (DOCX)

S1 Code R script used to reproduce the analyses and graphical outputs. (R)
