# Supplementary material for: Allometric equations for estimating tree volume and aboveground biomass of Acacia mangium Willd. on the Batéké Plateau, Republic of the Congo
Source: PLoS One. 2026 Sep 3;21(9):e0335578. doi: 10.1371/journal.pone.0335578 (PMC13541184; doi:10.1371/journal.pone.0335578)
Supplement: Supplementary file 1 [file pone.0335578.s005.docx]

**S1 Table. Tree height measurement methods comparison (repeated measures ANOVA)**

| **Site** | **Age**  **(months)** | **Mean DBH (cm)** | **Total height (Ht) of stand trees (m)** | | | | **Total length of felled tree (m)** |
| --- | --- | --- | --- | --- | --- | --- | --- |
|  |  |  | ***Position 1*** | ***Position 2*** | ***Position 3*** | ***Mean Position*** |  |
| **Site 1** | 66 | 12.7 ± 5.7 | 14.4 ± 3.5 | 14.4 ± 3.8 | 15.0 ± 4.2 | 14.6 ± 3.8 | 14.7 ± 3.8 |
| **Site 2** | 42 | 12.0 ± 4.4 | 10.4 ± 1.7 | 10.3 ± 1.7 | 10.3 ± 1.5 | 10.3 ± 1.6 | 10.4 ± 1.5 |
|  | 25 | 9.9 ± 2.8 | 8.6 ± 1.5 | 8.5 ± 1.3 | 8.1 ± 1.4 | 8.4 ± 1.4 | 8.5 ± 1.1 |
| **Site 3** | 25 | 9.7 ± 4.4 | 9.2 ± 1.9 | 9.3 ± 1.8 | 9.2 ± 1.7 | 9.2 ± 1.8 | 9.5 ± 1.5 |
|  | 25 | 9.5 ± 3.3 | 6.2 ± 1.2 | 6.4 ± 1.1 | 6.2 ± 1.0 | 6.3 ± 1.1 | 5.7 ± 1.0 |
| **Average** | | 11.0 ± 4.5 | 10.3 ± 3.7 | 10.3 ± 3.7 | 10.4 ± 4.0 | 10.3 ± 3.8 | 10.3 ± 3.9 |
| **repeated measures ANOVA** | | | p-value = 0.911 (> 0.05); F(3,159) = 0.178; ges = 0.000064 | | | | |

*Raw distributions were non-normal (Shapiro–Wilk, p ≤ 0.001 for all four methods), but normality was achieved after log transformation (p = 0.097-0.219). A repeated-measures ANOVA on log-transformed data showed no method effect (F(3,159) = 0.178, p = 0.911; generalized eta squared (ges) = 0.000064. These findings indicate that measures at Position 1, 2, 3, and Felled yield equivalent total height estimates*
